# An Ultra-Low-Noise, Low Power and Miniaturized Dual-Channel Wireless Neural Recording Microsystem

**DOI:** 10.3390/bios12080613

**Published:** 2022-08-08

**Authors:** Haochuan Wang, Qian Ma, Keming Chen, Hanqing Zhang, Yinyan Yang, Nenggan Zheng, Hui Hong

**Affiliations:** 1 Key Laboratory of Radio Frequency Circuit and System, Hangzhou Dianzi University, Hangzhou 310018, China; 2 Qiushi Academy for Advanced Studies, Zhejiang University, Hangzhou 310027, China; 3 College of Computer Science and Technology, Zhejiang University, Hangzhou 310027, China

**Keywords:** wireless neural recording, full-differential front-end structure, hierarchical microassembly technology, ultra-low noise, low-power system, system miniaturization

## Abstract

As the basic tools for neuroscience research, invasive neural recording devices can obtain high-resolution neuronal activity signals through electrodes connected to the subject’s brain. Existing wireless neural recording devices are large in size or need external large-scale equipment for wireless power supply, which limits their application. Here, we developed an ultra-low-noise, low power and miniaturized dual-channel wireless neural recording microsystem. With the full-differential front-end structure of the dual operational amplifiers (op-amps), the noise level and power consumption are notably reduced. The hierarchical microassembly technology, which integrates wafer-level packaged op-amps and the miniaturized Bluetooth module, dramatically reduces the size of the wireless neural recording microsystem. The microsystem shows a less than 100 nV/$\sqrt{\mathrm{Hz}}$ ultra-low noise level, about 10 mW low power consumption, and 9 × 7 × 5 mm${}^{3}$ small size. The neural recording ability was then demonstrated in saline and a chronic rat model. Because of its miniaturization, it can be applied to freely behaving small animals, such as rats. Its features of ultra-low noise and high bandwidth are conducive to low-amplitude neural signal recording, which may help advance neuroscientific discovery.

## 1. Introduction

Invasive neural recording, due to its closer proximity to neurons, can collect neural activity signals with high resolution, making it a powerful means of neuroscientific research [1,2,3,4]. Recently advanced electrode fiber arrays have been proposed to map and modulate deep brain activity by optical, electrical, and chemical means [5,6]. Although two-photon imaging can monitor neural activity signals with higher spatial resolution, it is difficult to miniaturize the device and faces significant barriers in clinical translation [7,8,9]. The electrical neural recording has an incomparable ultra-high resolution in temporal scale, is well established for basic science and clinical research, and there are more approaches to realize the miniaturization of equipment [10,11,12]. Miniaturizing electrical neural recording devices will produce less interference with biological activities and will facilitate the study of neural activity patterns in freely behaving small animals.

Significant progress has been made in invasive electrical neural recording devices. The brain–computer interfaces constructed with application-specific integrated circuits (ASIC) realize miniaturization, complete implantation, and multimodal integration [13,14,15]. However, all components are fabricated on a unified CMOS process, which cannot achieve high Q-value inductors and large-capacity capacitors, limiting the efficiency of wireless communication and energy harvesting in wireless neural recording devices. The coupling of analog and digital circuits on the same substrate also creates additional interference. Therefore, neural recording systems designed around ASIC usually have inferior noise performance and wireless communication efficiency, which restrict their application in neural activity research [16,17,18]. Systems integrated with discrete components usually have the advantages of low noise, high efficiency and high flexibility. They remain the primary tools in neuroscience research [19,20,21].

**Figure 1:**
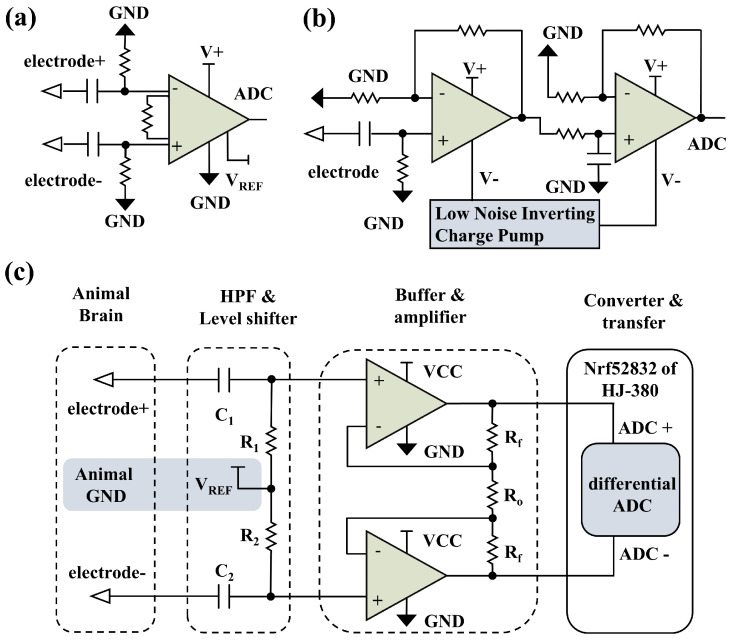
The comparison of front−end structures. (**a**) The front−end structure construction of instrumentation amplifier. (**b**) The front−end structure construction of cascade op−amps. (**c**) The dual op−amps full differential neural recording front−end structure we proposed.

Neural recording systems integrated with discrete components typically include a neural recording front-end circuit to pre-amplify the neural signals, and a wireless communication module to decrease the effects of animals’ free behavior. As the extracellular neural signals have ultra-low amplitudes (typically 50–500 μV), the recording front-end circuit requires a gain of more than 60 dB. The demand for low noise and high input impedance further increases the challenge of designing the neural recording front end, especially in power-constrained conditions [22,23]. The front-end circuit built with instrumentation amplifiers (Figure 1a) can support high gain and low noise requirements, and its simple structure facilitates the assembly of neural recording devices [24,25]. However, the large bandwidth beyond the requirements results in high power consumption and limits further miniaturization. front ends built with discrete op-amps (Figure 1b) typically require an input buffer stage to provide high input impedance, a separate gain stage to provide high gain levels, and a low-noise negative power chip to capture alternating neural signals [26,27,28]. The use of multi-stage circuits unfavorably amplifies the noise coupled between the circuit connection stages, and the low-noise negative power chips also increase the power consumption and area of the circuit. Therefore, the existing wireless neural recording systems designed by discrete components are usually bulky and far from reaching their size limit.

Further miniaturized, high-performance, and easy assembly wireless neural recording devices will meet the requirements of recording in freely behaving small animals. To reduce the dimensions and power consumption, it is important to construct a novel amplifier front-end circuit and select the appropriate communication schemes. In recent years, microassembly technology, including wafer-level packaged chips and miniaturized modules, has been widely used to improve system integration density and reduce the parasitic effects [29,30]. Employing microassembly technology to further reduce the size and improve the performance makes it possible to construct a miniaturized and high-performance wireless neural recording system.

**Figure 2:**
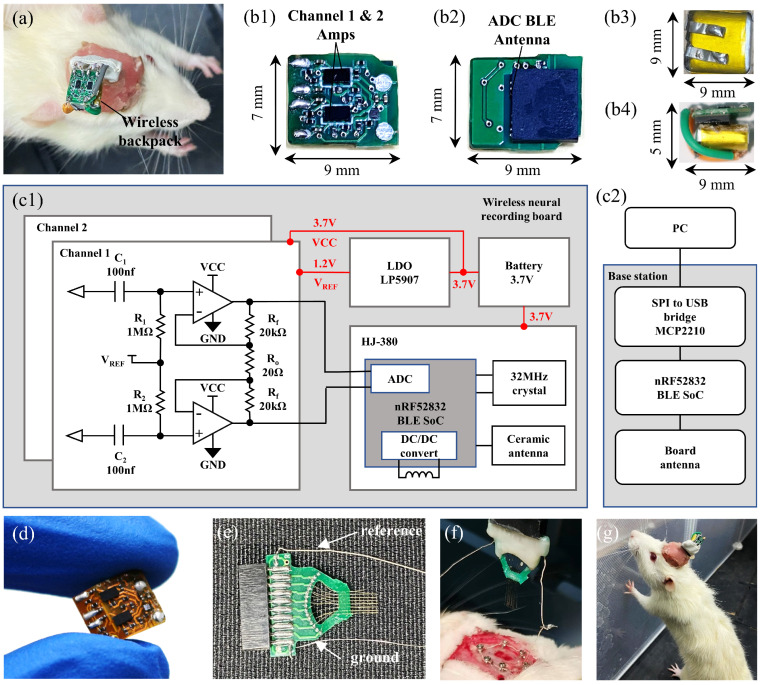
Ultra−low noise, miniaturized and lightweight dual−channel wireless neural recording microsystem, and the freely moving rats within this system. (**a**) Photograph of a rat assembly with the wireless neural recording microsystem through an implanted 16−channel rigid electrode array. (**b**) Photograph of components of the wireless neural recording microsystem: (**b1**) op−amps of neural signal amplification front end, (**b2**) miniaturized Bluetooth low−energy module, (**b3**) 30 mAh, 3.7 V lithium battery, (**b4**) wireless neural recording microsystem connected to the battery. (**c**) Schematic of the wireless neural recording system, (**c1**) schematic of the ultra−low−noise neural recording front end and the wireless neural recording microsystem, and (**c2**) schematic of the receiver base station. (**d**) The flexible substrate wireless neural recording microsystem. (**e**) Implantable 16−channel rigid electrode array. (**f**) Photograph of the electrode array implantation process. (**g**) Freely moving rat with the wireless neural recording microsystem.

This paper developed an ultra-low-noise, low power and miniaturized dual-channel wireless neural recording microsystem, as shown in Figure 2. With the dual op-amps, full differential amplifier front-end structure and employing the hierarchical microassembly technology, the miniaturized wireless neural recording microsystem was realized. We confirmed the recording capability of the wireless neural recording microsystem in saline and chronic rat model, and compared it with the wired neural recording system.

## 2. Materials and Methods

### 2.1. Ultra-Low-Noise and Low-Power Neural Recording Front End

For the reduction of components and power consumption, a full differential neural recording front end of dual op-amps is proposed, as shown in Figure 1c and Figure 2(c1). Two independent op-amps are used to form the fully differential amplifier structure. The positive input terminals of the two op-amps are used as differential signal inputs, providing an input impedance of 10${}^{9}$Ω order. The negative input and output terminals of the two op-amps constitute a feedback loop with resistors $R_{o}$ and $R_{f}$. The differential signal gain can be flexibly changed by adjusting the values of $R_{o}$ and $R_{f}$. The buffering and amplifying functions are achieved simultaneously by using two independent op-amps. The differential input structure and high input impedance make it possible to introduce a reference voltage through a high-pass filter at the input terminals. A low dropout regulator (LDO) is utilized as a reference voltage to lift the DC level of the signal, and the need for a low-noise negative power chip is eliminated.

**Figure 3:**
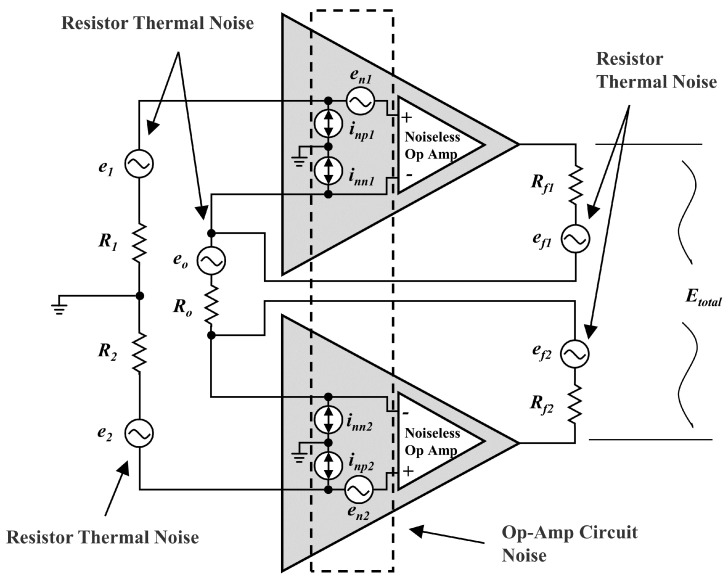
The equivalent noise circuit diagram of the dual op−amps full differential neural recording front end.

Figure 3 shows the equivalent noise circuit diagram of the dual op-amps full differential neural recording front end, where $e_{1}$, $e_{2}$, $e_{o}$, $e_{f1}$ and $e_{f2}$ represent the equivalent thermal noise of the resistors. $i_{nn1}$, $i_{nn2}$, $i_{np1}$ and $i_{np1}$ represent op-amps’ internally generated current noise. $e_{1}$ and $e_{1}$ are the internally generated voltage noise of op-amps. Since $R_{1}=R_{2}$, $R_{f1}=R_{f2}$, the output noises produced by the resistors’ equivalent thermal noise are shown as:

(1) $$E_{2}=E_{1}=e_{1}\left(1+2\frac{R_{f1}}{R_{o}}\right)=\sqrt{\int 4kTR_{1}df}\left(1+2\frac{R_{f1}}{R_{o}}\right)$$

(2) $$E_{o}=e_{o}\frac{2R_{f1}}{R_{o}}=\sqrt{\int 4kTR_{o}df}\frac{2R_{f1}}{R_{o}}$$

(3) $$E_{f1}=E_{f2}=e_{f1}=\sqrt{\int 4kTR_{f1}df}$$

where *k* is Boltzmann’s constant (1.38 × 10${}^{-23}$ j/K), and *T* is the absolute temperature in Kelvin (K). In general, $i_{nn1}=i_{nn2}$, $i_{np1}=i_{np2}$ and $e_{1}=e_{2}$, the output noises produced by the op-amps’ internal equivalent noise sources are shown as:

(4) $$E_{n1}=E_{n2}=\int e_{n1}\left(1+2\frac{R_{f1}}{R_{o}}\right)df$$

(5) $$E_{nn1}=E_{nn2}=\int i_{nn1}\left[R_{f1}//\left(R_{o}+R_{f2}\right)\right]df$$

(6) $$E_{np1}=E_{np2}=\int i_{np1}R_{1}\left(1+2\frac{R_{f1}}{R_{o}}\right)df$$

The output noise is analyzed using the principles of superposition, and each of the noise sources is isolated. The gain of the circuit can be expressed as $\left(1+2\frac{R_{f}}{R_{o}}\right)=A$, and the total noise of the circuit is shown as:

(7) $$\begin{array}{c} E_{\mathrm{total}\;}=\sqrt{\begin{array}{c} E_{1}{}^{2}+E_{2}{}^{2}+E_{f1}{}^{2}+E_{f2}{}^{2}+E_{o}{}^{2}\\ +E_{n1}{}^{2}+E_{n2}{}^{2}+E_{nn1}{}^{2}+E_{nn2}{}^{2}+E_{np1}{}^{2}+E_{np2}{}^{2} \end{array}}\\ =\sqrt{\int [\begin{array}{c} 4kT\left(2R_{1}A^{2}+2R_{f}+R_{o}{\left(A-1\right)}^{2}\right)\\ +2e_{n1}{}^{2}A^{2}+2i_{nn1}{}^{2}{\left(R_{f1}//\left(R_{o}+R_{f2}\right)\right)}^{2}+2i_{np1}{}^{}2R_{1}{}^{2}A^{2} \end{array}]df} \end{array}$$

The noise of resistors is constant at related frequencies. The input-referred noise of op-amps can be expressed as the combination of white and 1/f noise. Therefore, the total noise in Equation (7) can be simplified as:

(8) $$E_{\mathrm{total}\;}=\sqrt{\begin{array}{c} ENB\left(4kT\left(2R_{1}A^{2}+2R_{f}+R_{o}{\left(A-1\right)}^{2}\right)\right)\\ +2i_{w}{}^{2}\left[{\left(R_{f1}//\left(R_{o}+R_{f2}\right)\right)}^{2}+R_{1}^{2}A^{2}\right]\left(f_{\mathrm{inc}\;}\ln\frac{f_{H}}{f_{L}}+ENB\right)\\ +2e_{w}{}^{2}A^{2}\left(f_{\mathrm{enc}\;}\ln\frac{f_{H}}{f_{L}}+ENB\right) \end{array}}$$

where $i_{w}$ is the white current noise specification (spectral density in $A/\sqrt{Hz}$), $f_{inc}$ is the current noise corner frequency, $e_{w}$ is the white voltage noise specification (spectral density in $V/\sqrt{Hz}$), and $f_{enc}$ is the voltage noise corner frequency. ENB is the effective noise bandwidth.

According to Equation (8), the input resistors $R_{1}$, $R_{2}$ and feedback resistor $R_{o}$ should be reduced to decrease the circuit noise. Selecting op-amps with lower equivalent noise and strictly limiting the bandwidth to the required range is key to reducing front-end circuit noise. The differential structure reduces the RF interference introduced through signal paths, which improves the noise performance of the circuit. This structure with two independent op-amps makes it possible to realize better trade-offs among gain, bandwidth, noise, power consumption, and package area.

### 2.2. Hierarchical Microassembly of Miniaturized Wireless Neural Recording Microsystem

Figure 2(c1) shows the schematic of the dual-channel full differential wireless neural recording microsystem. The input resistors $R_{1}$ and $R_{2}$ are 1 MΩ, with 100 nF capacitors $C_{1}$ and $C_{2}$ forming the high-pass filters of 1.59 Hz, to eliminate voltage drift generated by the electrode–tissue interface. For the reduction of front-end circuit noise, the dual-channel op-amp chip (opa2376, Texas Instruments Inc., Dallas, TX, USA) with a noise floor of 7.5 nV/$\sqrt{\mathrm{Hz}}$ is selected. With $R_{o}$ of 20 Ω and $R_{f}$ of 20 kΩ, a 66 dB gain is achieved, and the bandwidth is limited to 6.5 kHz. This bandwidth covers the frequency range of local field potentials (LFP) and most spikes. The LDO (LP5907, Texas Instruments Inc., Dallas, TX, USA) generates a reference voltage of 1.2 V, and the DC level of the collected signal is raised to 1.2 V through the high-pass filters at the input terminal. Finally, the front-end circuit with a 3 mm${}^{2}$ area and the 1.5 mW power consumption is realized.

For further miniaturization of the wireless neural recording system, a miniaturized Bluetooth module (HJ-380, Tangshan Hongjia Electronic Technology, Tangshan, China) on the Bluetooth chip (nRF52832, Nordic Semiconductor Inc., Trondheim, Norway) is adopted. The dual-channel differential ADC of nRF52832 samples the amplified neural signals at a sampling rate of 20 ksps. A 10 μH inductor is integrated into the module for the DC/DC circuit, which decreases the module power to 3 mA. The modulated data of ADC are then sent to the base station through the internal ceramic antenna. The microsystem is constructed on a 0.8 mm-thick high-density FR-4 printed circuit board, and automatic alignment is used for microdevices soldering with high precision. Figure 1b shows the photograph of the realized miniaturized dual-channel microsystem. The overall size of the microsystem is 7 × 9 × 2.6mm${}^{3}$, and the weight is 257 mg (955 mg with battery). The microsystem is powered by a 3.7 V lithium battery (30 mAh, 9 × 9 mm${}^{2}$), and can work continuously for 1.5 h.

### 2.3. Base Station and Software

The base station is constructed by a Bluetooth development board on nRF52832. The system uses BLE5.0 protocol for wireless communication, and the connection interval is 7.5 ms. The recorded neural signal data are sent to the base station in real time. The communication rate between the microsystem and the base station is 800 kbps, and the neural signal data and channel tags are transmitted. For the prevention of data loss caused by the interference of Bluetooth communication, a cache area of 5 KB is allocated inside the Bluetooth chip to store the data stream. The serial peripheral interface (SPI) to universal serial bus (USB) conversion module reads the base station data at a rate of 3 Mbps and sends them to the computer. Software is developed based on Qt to receive and transcode the data in real time. After transcoding, the neural signal waveform is drawn on the GUI during experiments, and the data are saved in CSV format simultaneously. The data saved are further analyzed by MATLAB software.

### 2.4. Chronic Rat Model Experiment and Comparison with the Commercial Wired Recording System

**Figure 4:**
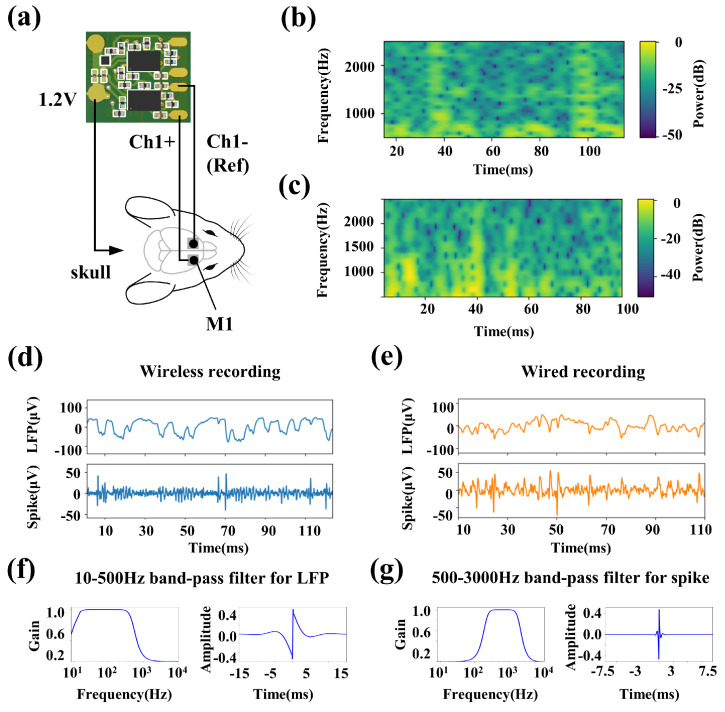
The waveforms comparison of wireless recording microsystem and apollo II wired recording system in vivo. (**a**) Schematic of the recording sites and the electrode connections. (**b**) The multi−taper spectrograms of recorded spikes by the wireless recording microsystem. (**c**) The multi−taper spectrograms of recorded spikes by the Apollo II wired recording system. (**d**) The recorded spontaneous LFPs and spikes by the wireless recording microsystem. (**e**) The LFPs and spikes by the Apollo II wired recording system. (**f**) The specification of 10–500 Hz second−order Butterworth band-pass filter and its step response. (**g**) The specification of 500–3000 Hz third−order Butterworth band-pass filter and its step response.

The wireless neural recording microsystem is validated in vivo. A rat of 520 g is selected for the experiment. To implant the electrode array, the rat is anesthetized with 1.3 mL of 2% sodium pentobarbital by intraperitoneal injection. A 16-channel rigid electrode array (25 μm diameter tungsten) is implanted into the motor cortex (M1) of the right hemisphere via fenestration of the skull and dura, and the reference electrode (25 μm diameter tungsten) is implanted into the M1 of the left hemisphere, as shown in Figure 2e,f and Figure 4a. Cranial nails are implanted around the fenestration as a ground electrode, and the ground electrode is connected through a silver wire. Dental cement is used to fix the electrode array on the skull. The rat is used for the neural recording experiment after recovering for more than one week. A commercial wired neural recording system (Apollo II, China) is used for the comparison of the wireless neural recording microsystem. After the wireless neural recording microsystem experiment, the same electrode array is connected to the wired neural recording system and the same experiment is repeated. The wireless and wired rat recording experiments are performed within one hour, to minimize the effect of time variation on the quality of the recorded signal.

## 3. Results

### 3.1. High-Performance and Miniaturized Neural Recording Microsystem

**Figure 5:**
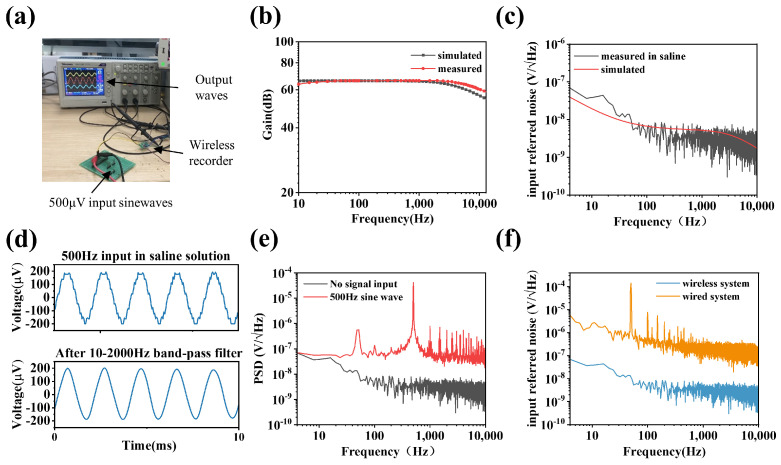
The electrical properties of wireless neural recording microsystem. (**a**) The transfer function test environment. (**b**) Transfer function measured and simulated of the dual op−amps full differential neural recording front end. (**c**) Input referred noise measured of the wireless neural recording microsystem in saline solution and compared with the simulation. (**d**) The waveform of recorded 500 Hz, 2 mV signal injected in the saline by the wireless neural recording microsystem using 35 μm diameter tungsten electrodes, and waveform after filtering out high−frequency interference. (**e**) Power spectral density in saline with or without 500 Hz input signal. (**f**) Input referred noise comparison of wireless recording microsystem and Apollo II wired recording system.

Figure 5a shows the test environment for the transfer function. The signal generator generated a 500 mV signal and then obtained a 500 μV input signal through a resistive subdivision. The input sine waves swept from 10 Hz to 10 kHz. The transfer function of the ultra-low noise and miniaturized neural recording front end is shown in Figure 5b. The measured gains ranged from 59–66.1 dB (simulated 54–66.1 dB), and the gain at 1 kHz is about 66 dB. The 3 dB bandwidth of the front end spanned from 10 Hz to 6.5 kHz. This bandwidth range can cover the LFP and most spikes.

A saline test platform is performed to evaluate the noise performance of the wireless neural recording microsystem. The tungsten electrodes are directly connected to the microsystem’s input end, and the tips of the electrodes are immersed in saline. The impedance of the electrodes is less than 2.5 Ω in the working frequency band of 10 Hz–100 kHz. The noise waveform data collected by the receiver is processed by MATLAB and divided by the gain of the front-end circuit. The input reference noise spectrum is obtained as shown in Figure 5c. The system exhibits a less than 100 nV/$\sqrt{\mathrm{Hz}}$ ultra-low noise level at 10 Hz–10 kHz, which is consistent with the simulation results.

We verify its signal acquisition ability in saline. A synthetic sine wave of 2 mV${}_{p-p}$ and 500 Hz is injected into saline. The waveform data received by the base station are shown in Figure 5d. Figure 5e compares the power spectral density with or without input signals. The signal-to-noise ratio (SNR) reaches about 30 dB when the 2 mV${}_{p-p}$ sine wave is injected. In order to accurately compare the noise performance of wireless recording microsystems and the wired recording system, we repeat the noise tests on the wired recording system under the same scenario. Figure 5f shows the comparison of input reference noise spectrums; the 1 m-long wire of the wired recording system introduces a significant power frequency interference. At the same time, the noise level is also above 5 μV/$\sqrt{\mathrm{Hz}}$, without considering the power frequency interference.

### 3.2. In Vivo Neural Recording Experiment

Figure 4a shows the electrode connections of the wireless recording microsystem. For comparison with the wired neural recording device, the two electrodes of channel-1 are connected to the bilateral M1 area of the rat, and the 1.2 V reference voltage is connected to the electrode array ground (which is connected to the cranial nail implanted in the skull by silver wire). The rat moves freely in a square field of 40 × 40 cm${}^{2}$. We successively implement the wireless recording microsystem and the Apollo II wired recording system to record neural signals generated during free movement in rats.

The recorded neural signals are shown in Figure 4d,e. The LFP signals are obtained through a 10–500 Hz second-order Butterworth band-pass filter, and the spikes are obtained through a 500–3000 Hz third-order Butterworth band-pass filter. The specifications of band-pass filters and their corresponding step responses are shown in Figure 4f,g. The results show that the wireless recording microsystem exhibits a similar performance to the wired recording system in LFPs recording. Figure 4f,g show the multi-taper spectrograms of spikes in Figure 4d,e, respectively. The wireless recording microsystem shows a lower high-frequency interference and is conducive to extracting clear spikes.

## 4. Discussion

Through the reconstruction of the front-end circuit structure and the application of microassembly technology, an ultra-low-noise, low power consumption and miniaturized dual-channel wireless neural recording microsystem is developed. The power consumption of a single channel is 1.5 mW, and the system’s noise level is less than 100 nV/$\sqrt{\mathrm{Hz}}$, which are realized by the fully differential front end with dual op-amps. The microassembly technology realizes the miniaturization of the wireless recording microsystem; the overall size is 9 × 7 × 5 mm${}^{3}$. Its weight with the battery is then reduced to 955 mg. The neural recording capacity has been verified in a chronic rat model. Its miniaturized size and weight can be used in the neural recording of rats, and has little influence on their free behavior. With the ultra-low-noise feature, it can clearly acquire neural signals.

Table 1 lists the features of the microsystem and other wireless recording systems integrated with discrete components. Compared with the reported smallest wireless recording system, the volume is reduced by 86%, and the weight is reduced by 66%. The noise and power consumption are the smallest in the reported work. The ultra-thin and lightweight characteristics of the polyimide substrate reduced the weight of the microsystem to 810 mg (Figure 2d), but the solder balls are prone to fatigue fracture and cause circuit failure in actual use. The additional reinforcing board is required to enhance the reliability of the flexible substrate board, which will increase the system’s weight and result in a limited improvement in overall weight.

The front-end circuit proposed for bioelectrical signal recording shows more flexibility in the trade-off between area, power consumption, gain, and bandwidth. It is beneficial to multiple bioelectrical signal acquisition applications. The power consumption can be further reduced by reducing the noise limit and gain bandwidth. For example, in some neural signal decoding tasks, only the 0.1–500 Hz LFP signal is required. The noise limit can be increased to over 5 μV/$\sqrt{\mathrm{Hz}}$, and the power consumption can be reduced by 1–2 orders of magnitude [31,32,33], which will contribute to an increase in the number of channels, or a reduction in power consumption, further realizing the system miniaturization.

## 5. Conclusions

An ultra-low-noise, low power consumption and miniaturized dual-channel wireless neural recording microsystem with fully differential neural recording front end and microassembly technology is developed. The overall size of the microsystem is 9 × 7 × 5 mm${}^{3}$, and the total weight is 955 mg. Compared with the reported smallest wireless neural recording system, the microsystem volume is reduced by 86%, and the weight is reduced by 66%. The input referred noise of the microsystem is less than 100 nV/$\sqrt{\mathrm{Hz}}$, and the power consumption is about 10 mW. Its neural recording ability is confirmed in saline and a chronic rat model, and compared with a commercial wired neural recording system. The experiments show that the microsystem has little effect on the natural behavior of rats. Its miniaturization and light weight enable it to be used in small biological neural recording scenes, which is conducive to promoting neuroscience research.

## Figures and Tables

**Table 1:** Comparison of the microsystem features and other wireless recording systems fully validated in vivo.

	TBSI [26]	PennBMBI [27]	WAND [16]	BLE Recording [28]	Wireless Bidirectional [21]	This Work
Year	2011	2015	2019	2021	2022	2022
Size (mm${}^{3}$)	22 × 22 × 22	56 × 36 × 13	36 × 33 × 15	15 × 15 × 12	19.9 × 18.1 × 6.6	9 × 7 × 5
Weight (g)	4.5	-	7.4 (board) 17.95 (total)	3.9 (total)	2.8	0.257 (board) 0.955 (total)
Power consumption (mW)	-	290	172	28.6	62	∼10
Input referred noise (μV/$\sqrt{\mathrm{Hz}}$)	10	4.7	26	3	2.4 ^1^	<0.1
Number of channels	15	4	128	1	8	2
Sampling rate (ksps)	-	21	1	10	20	20
ADC resolution (bits)	-	12	15	12	16	12

^1^ The noise of commercial brain–computer interface chip RHS2116.

## Data Availability

Not applicable.
